# Menstruation-Related Disorders—Dysmenorrhea and Heavy Bleeding—as Significant Epiphenomena in Women With Rheumatic Diseases

**DOI:** 10.3389/fphar.2022.807880

**Published:** 2022-02-04

**Authors:** Martina Orlandi, Silvia Vannuccini, Khadija El Aoufy, Maria Ramona Melis, Gemma Lepri, Gianluca Sambataro, Silvia Bellando-Randone, Serena Guiducci, Marco Matucci Cerinic, Felice Petraglia

**Affiliations:** ^1^ Department of Experimental and Clinical Medicine, University of Florence and Division of Rheumatology AOUC and Scleroderma Unit, Florence, Italy; ^2^ Obstetrics and Gynecology, Department of Maternity and Infancy, AOU Careggi Florence, Florence, Italy; ^3^ Artroreuma S.R.L., Rheumatology Outpatient Clinic Associated with the National Health System, Catania, Italy; ^4^ Unit of Immunology, Rheumatology, Allergy and Rare Diseases (UnIRAR), IRCCS San Raffaele Hospital, Milan, Italy

**Keywords:** heavy menstrual bleeding, menstruation disorders, dysmenorrhea, dyspareunia, rheumatic and musculoskeletal disease, gynecological diseases, rheumatic disease

## Abstract

**Background:** In women with rheumatic diseases (RDs) menstruation-related disorders have never been investigated. The aim of this study was to evaluate gynecological symptoms/disorders in fertile age women with RDs.

**Materials and methods:** All patients (n = 200) filled up a self-administered questionnaire on their gynecological history, menstrual cycle pattern, menstrual-related symptoms, and quality of life (QoL). The RD group was then compared to a control group of 305 age-matched fertile age women.

**Results:** Among patients with RDs, 58% had arthritis, 40% connective tissue diseases (CTDs), and 1.5% systemic vasculitis. No differences were observed between CTDs and arthritis, except for a family history of HMB which was more common among women with CTDs (*p* < .01). When compared to controls, women with RDs reported more frequent heavy menstrual bleeding (HMB) during adolescence (51.7 and 25.4%, respectively; *p* = .0001) and adult life (37.7 and 25.9%, respectively; *p* = .0065). Also, dysmenorrhea in adolescence was significantly more common among cases (55.6 and 45.4%, respectively; *p* = .0338). Gynecological pain (dysmenorrhea, non-menstrual pelvic pain, dyspareunia, dysuria, and dyschezia) in patients with RDs was more frequent than in controls (*p* = .0001, .0001, .0001, .0001, .0002, respectively). Considering women who reported moderate and severe symptoms in RDs, dysmenorrhea and dyspareunia remain significantly more frequent in women with RDs than in controls (*p* = .0001; *p* = .0022; respectively). QoL scores were significantly reduced in women with RDs, either in physical (*p* = .0001) and mental domains (*p* = .0014) of short-form 12.

**Conclusion:** Women affected by RDs frequently presented menstruation-related disorders; thus, female patients with RDs should be questioned about gynecological symptoms and referred to the gynecologist for an accurate evaluation.

## Introduction

Rheumatic diseases (RDs) affect significantly more women than men, and for this reason, gynecological disorders may have an additional negative impact on women’s health (Krasselt and Baerwald, 2019).

From menarche to menopause, gynecological and reproductive health is an essential part of well-being among women. In fertile age, physical, mental, and social aspects are largely influenced by sex hormonal changes during the menstrual cycle, possibly leading to pathological conditions (Matteson and Zaluski, 2019; Critchley et al., 2020a). The most common gynecological symptoms associated with menstruations are dysmenorrhea (pain related to the uterine contractility) (Bernardi et al., 2017) and bleeding (shedding of endometrial cells and menstrual blood). These problems, according to their intensity and volume (severe dysmenorrhea and heavy menstrual bleeding (HMB)) (Critchley et al., 2020b), may cause a relevant impact on QoL. Moreover, they may be also early signs of menstruation-related disorders, such as uterine fibroids, endometriosis, and adenomyosis (Maybin and Critchley, 2015).

Gynecological problems represent an important need to be considered by the rheumatologist. Among patients with RDs, the disturbance of gynecological health may be ascribed to not only several factors such as chronic inflammation, hormonal imbalance, and drug effects but also psychological and cultural issues (Østensen et al., 2015). A number of evidence have been published on pregnancy outcomes and recommendation on preconception care (Marder and Johnson, 2020; Sammaritano et al., 2020). Regarding gynecological issues, mostly sexuality and vaginal symptoms, related to dryness, have been considered (Østensen, 2017). However, limited data are available on menstruation-related disorders in RDs (Haga et al., 2005). Recently, the key role of a rheumatologist in the management of RDs and their interaction with reproductive health has been recognized (Sammaritano et al., 2020). In clinical practice, a better understanding of the underlying phenomena involved in menstruation (pain and bleeding) and other menstruation-related disorders may allow to achieve the goal of personalized care. Thus, the aim of this study was to describe gynecological symptoms among fertile age women with RDs and their impact on Qol.

## Materials and Methods

A monocentric, cross-sectional observational study was conducted in the Rheumatology Department, in collaboration with the Gynecology and Obstetrics Unit of Careggi Hospital in Florence from June to December 2020. The inclusion criteria were the diagnosis of RDs [connective tissue diseases (CTDs), arthritis, and spondyloarthritis], female gender, and fertile age. The study was approved by the local Ethics Committee (protocol number 15603_oss, Florence, 2020; February 26th).

All enrolled patients were investigated about their gynecological history and symptoms during their routinely visit in the RD outpatient clinic. A self-administered anonymized questionnaire, elaborated by the gynecology team, was administered, including all the principal aspect of gynecological and reproductive health (Supplementary Material). Multiple choices or yes/no questions were asked in order to improve accuracy. The first part of the questionnaire was about gynecological family history of HMB, dysmenorrhea, or chronic pelvic pain and the first 1,000 days of life, including the mode and gestational age at birth, birth weight, and breastfeeding or formula feeding. Next, data on menarche, presence of dysmenorrhea, and HMB during adolescence and adulthood were asked. For the evaluation of gynecological pain (dysmenorrhea, non-menstrual pelvic pain, dyspareunia, dysuria, and dyschezia) the Numeric Rating Scale was chosen because of its reliability, accuracy, and simplicity of use. It is a point scale numbered from 0 (no pain) to 10 (maximum pain), where the patient attributes the value based on to the proven experience. The pain is considered as mild for 1–3 points, moderate for 4–7 points, and severe for 8–10 points. The presence of vaginal and sexual symptoms, in terms of sicca syndrome, was also addressed. Previous obstetric history and possible fertility issues were also assessed. Gynecological diseases such as endometriosis, adenomyosis, and uterine fibroids, and systemic comorbidities, such as autoimmune thyroiditis, endocrine/metabolic diseases, inflammatory bowel diseases, and mental health disorders were investigated. Finally, QoL was assessed in all patients with the Short-Form (12) Health Survey (SF-12, reduced size version of the SF-36) (Gandek et al., 1998). This questionnaire is widely used since it produces similar results for physical and mental health scores with far less respondent burden for producing scores of overall mental and physical well-being.

Data collected through the questionnaires were internally compared among women with RDs, on the basis of pathogenetic similarities between those with CTDs and those with arthritis. A comparison was also made between the RD group and a control group of 305 age-matched fertile age women undergoing gynecological routinely checkup.

### Statistical Analysis

All demographics and gynecological data were reported in an electronic database, protected by a password to satisfy current privacy standards. Statistical analysis was performed using IBM SPSS Statistics software, version 22 (IBM Corporation, Armonk, NY, United States). Descriptive statistics was performed: continuous variables were expressed as mean (±standard deviation, SD), whereas for categorical data, absolute and relative frequencies were calculated. In order to evaluate the distribution of continuous parameters in each group, the normal distribution and homoscedasticity were tested using the Shapiro–Wilk test and the Bartlett test, respectively. To evaluate the association between continuous parameters and patient groups, Student’s *t* test, Satterthwaite *t* test, or the Mann–Whitney test were used, according to the results of the normal distribution and the homoscedasticity test. In order to evaluate the association between categorical variables and patients’ groups a chi-squared test or Fisher’s exact test were used. A *p*-value < 0.05 was considered statistically significant.

## Results

From June 3rd to 30 December 2020, a total of 236 women with RDs were consecutively enrolled in the study from the Rheumatological Outpatient Clinic of AOU Careggi in Florence. Finally, 200 of them definitively participated in the study. In this population, RDs were distributed as follows: 81 CTDs (40.5%), 116 arthritis (58%), and three vasculitis (1.5%) (Table 1). The mean overall age was 39.1 ± 8.7 years.

**TABLE 1:** Detailed rheumatological diagnosis of the patients included in the study.

Rheumatic diagnosis		Frequency, n (%) tot = 200
**CTDs** (40.5%), n = 81	SLE	6 (3%)
	SS	8 (4%)
	SSc	30 (15%)
	Antiphospholipid syndrome	1 (2%)
	Inflammatory myopathies	3 (1.5%)
	Overlap syndrome	8 (4%)
	UCTD	25 (12.5%)
**Arthritis** (58%), n = 116	Spondyloarthritis	24 (12%)
	Psoriatic arthritis	22 (11%)
	RA	67 (33.5%)
	Seronegative arthritis	3 (1.5%)
**Systemic vasculitis** (1.5%), n = 3	Behcet’s syndrome	2 (1%)
	Churg–Strauss syndrome	1 (0.5%)

CTDs: connective tissue diseases; SLE: systemic lupus erythematosus; UCTDs: Undifferentiated connective tissue diseases; RA: rheumatoid arthritis; SS: Sjögren’s syndrome, SSc: systemic sclerosis.

### Gynecological Data From Women With RDs

Gynecological data of patients with RDs are summarized in Table 2. In menstrual family history, HMB and dysmenorrhea were reported in more than half of the cases, whereas a history of chronic pelvic pain was reported in only 7% of patients. Regarding the first 1,000 days of life of women with RDs, caesarean birth was reported in 9%, and preterm birth in 8% of cases. The mean weight at birth was 3.269 ± 0.614 kg. A total of 119 patients were breastfed (63%), and in 39 patients, feeding was artificial (21%), and mixed in 31 (16%).

**TABLE 2:** Gynecological data of enrolled population.

Gynecological data, n/tot response	RDs (n = 200)	CTDs (n = 81)	Arthritis (n = 116)	*p*-value
Age (years)	39.08 ± 8.69	41.42 ± 7.03	37.29 ± 9.40	0.0040^a^
Age at RD diagnosis (years)	32.35 ± 10.30	34.71 ± 10.34	30.44 ± 11.09	0.4448
RD duration	7.88 ± 6.9	7.17 ± 6.74	7.93 ± 6.80	0.8434
Gynecological family history				
HMB	49/83 (59.03%)	25/36 (69.93%)	21/44 (47.73%)	0.0506^a^
Dysmenorrhea	46/83 (55.42%)	21/36 (58.83%)	23/44 (52.17%)	0.5878
Chronic pelvic	6/83 (7.22%)	5/35 (14.43%)	1/44 (2.22%)	0.0852
First 1,000 days				
Caesarean birth	17/189 (8.99%)	6/76 (7.77%)	11/110 (9.99%)	0.6243
Preterm birth	15/186 (8.06%)	5/73 (6.66%)	10/110 (8.88%)	0.5883
Mean weight at birth (g)	3.269 ± 0.614/101	3,321.4 ± 742.12/42	3,235.4 ± 506.37/57	0.5192
Breastfeeding	119/189 (62.96%)	46/75 (61.05%)	73/121 (65.49%)	0.4966
Formula feeding	39/189 (20.63%)	14 (18.87%)	23 (21.09%)	0.4966
Mixed feeding	31/189 (16.40%)	15 (19.98%)	15 (13.32%)	0.4966
Gynecological history in adolescence				
Menarche (years)	12.3 ± 2 years/192	12.45 ± 1.49/77	12.42 ± 1.70/112	0.4229
Dysmenorrhea	108/194 (55.67%)	44/78 (56.61%)	63/113 (55.5%)	0.9282
HMB	101/195 (51.79%)	41/79 (52.17%)	59/113 (52.17%)	0.9659
Current characteristics of menstrual cycle				
Dysmenorrhea	130/183 (71%)	51/70 (72.8%)	78/110 (71%)	0.2145
Mild	49/130 (37.7%)	14/51 (27.4%)	34/78 (43.6%)	-
Moderate	65/130 (50%)	28/51 (54.9%)	37/78 (47.4%)	-
Severe	16/130 (12.3%)	9/51 (17.6%)	7/78 (8.9%)	-
HMB	71/188 (37.76%)	33/76 (43.4%)	37/108 (34.2%)	0.2755
Fertility and reproductive health				
Time-to-pregnancy > 1 year	16/162 (9.87%)	7/63 (11.1%)	7/90 (7.77%)	0.4673
Previous miscarriage/s	40/131 (30.53%)	22/52 (42.18%)	18/77 (23.31%)	0.0199^a^
Previous pregnancy/s	107/191 (56%)	42/75 (55.5%)	64/113 (56.61%)	0.7987
Previous vaginal delivery	70/182 (38.5%)	23/31 (74.2%)	46/58 (78.81%)	0.4247
Previous caesarean section	27/182 (14.8%)	13/31 (41.07%)	14/57 (24.42%)	0.1023
Pain symptoms				
Non-menstrual pelvic pain	49/187 (26.2%)	22/83 (26.5%)	26/111 (23.4%)	0.3103
Mild	33/49 (67.3%)	-	-	-
Moderate	4/49 (28.6%)	-	-	-
Severe	2/49 (4.1%)	-	-	-
Dysuria	35/188 (18.6%)	12/74 (16.2%)	22/111 (19.8%)	0.5353
Mild	23/57 (40.3%)	-	-	-
Moderate	11/23 (47.8%)	-	-	-
Severe	1/23 (4.3%)	-	-	-
Dyschezia	35/188 (18.6%)	17/79 (21.5%)	18/108 (16.7%)	0.4357
Mild	22/35 (62.8%)	-	-	-
Moderate	10/35 (28.6%)	-	-	-
Severe	3/35 (8.6%)	-	-	-
Sicca symptoms and sexual dysfunction				
Vaginal dryness	67/187 (35.8%)	28/74 (37.74%)	37/111 (33.3%)	0.5588
Mild	46/67 (68.6%)	-	-	-
Moderate	17/67 (1%)	-	-	-
Severe	4/67 (2.13%)	-	-	-
Vaginal burning	54/187 (28.9%)	21/74 (28.3%)	32/110 (28.86%)	0.9167
Mild	42/54 (77.8%)	-	-	-
Moderate	10/54 (18.5%)	-	-	-
Severe	2/187 (3.7%)	-	-	-
Recurrent vaginal infections	35/180 (19.44%)	13/73 (17.76%)	21/104 (19.98%)	0.7470
Dyspareunia	72/184 (39.13%)	29/72 (39.96%)	41/108 (37.74%)	0.7188
Mild	39/72 (54.2%)	-	-	-
Moderate	27/72 (37.5%)	-	-	-
Severe	6/72 (8.3%)	-	-	-
External vaginal pain	27/65 (41.5%)	9/36 (24.42%)	18/54 (33.3%)	0.7366
Deeper pain	22/65 (33.8%)	10/37 (26.64%)	11/55 (19.98%)	-
Both	16/65 (24.6%)	7/37 (18.87%)	8/55 (14.43%)	-
Gynecological comorbidities				
Uterine fibroids	46/193 (23.83%)	19/75 (25.3%)	27/106 (23.3%)	0.4370
Endometriosis	20/200 (10%)	8/81 (9.9%)	11/116 (9.48%)	0.9220
PCOS	46/182 (25.27%)	10/74 (13.32%)	14/106 (13.32%)	0.9526
Comorbidities	71/161 (44.09%)	36/69 (52.17%)	35/89 (38.85%)	0.1073
Other autoimmune disorders	39/157 (24.8%)	26/39 (37.7%)	13/88 (14.7%)	0.0010^a^
Autoimmune thyroiditis	25/153 (16.33%)	11/62 (17.76%)	14/88 (15.54%)	0.7668
Endocrine/metabolic diseases	17/168 (10.11%)	6/66 (8.88%)	10/99 (9.99%)	0.829
Inflammatory bowel diseases	27/166 (16.26%)	13/65 (19.98%)	14/98 (14.43%)	0.3366
Mental health disorders depression	8/166 (4.81%)	3/69 (4.44%)	5/99 (5.55%)	0.1749
Anxiety	12/166 (7.22%)	7/65 (11.1%)	5/98 (5.55%)	1.0000
Quality of life _SF-12				
PCS-12	41.57 ± 12.47	39.09 ± 10.86	43.09 ± 13.17	0.0706
MCS-12	44.76 ± 11.23	42.40 ± 11.63	46.15 ± 10.92	0.0633

* HMB, heavy menstrual bleeding; PCOS, polycystic Ovary Syndrome; PCS-12, physical component score; MCS-12, mental component score. *p < 0.05.

Concerning gynecological history and the menstrual pattern*,* their first period was at 12.3 ± 2.0 years. In adolescence, 56% experienced dysmenorrhea and 52% of patients had HMB. Moreover, with regard to current symptoms, 71% of patients presented dysmenorrhea: mild in 38%, moderate in 50%, and severe in 12%. HMB was reported in 37% of patients. Furthermore, non-menstrual pelvic pain was reported in 26% of women, and in the majority of patients, the disorder was mild. Urinary pain was found in 19% of patients, mainly moderate or mild. Overall, 19% of patients had dyschezia, but it was mild in the majority of patients. Dyspareunia was present in 39% of patients, mild or moderate in the majority of cases. Dyspareunia was characterized by external, superficial vaginal pain in 41% of patients, and deeper pain was referred in 34%, while 25% suffered from both.

Vaginal symptoms were frequently reported: 36% of patients referred vaginal dryness, described as mild by the majority of patients. Vaginal burning was reported in 29%, mostly mild. Recurrent vaginal infections were referred by 19% of the patients.

Regarding reproductive health, fertility issues were reported in 10% of patients and almost 31% of patients experienced miscarriage, at least once. Otherwise, almost half of the patients had a pregnancy at least once (56%), and the majority had a spontaneous delivery (38.5%). In addition, uterine fibroids were reported in 24% of patients, endometriosis in 10%, and 25% of patients had polycystic ovary syndrome (PCOS).

Also, non-gynecological comorbidities were frequently reported. In particular, 44% had other autoimmune diseases (thyroiditis, diabetes, and celiac disease), 16% endocrinology diseases, 10% inflammatory bowel diseases, and 16.3% mental health diseases (anxiety, depression, anorexia, psychosis, and other). Depression and anxiety were diagnosed in 5 and 7%, respectively.

**FIGURE 1:**
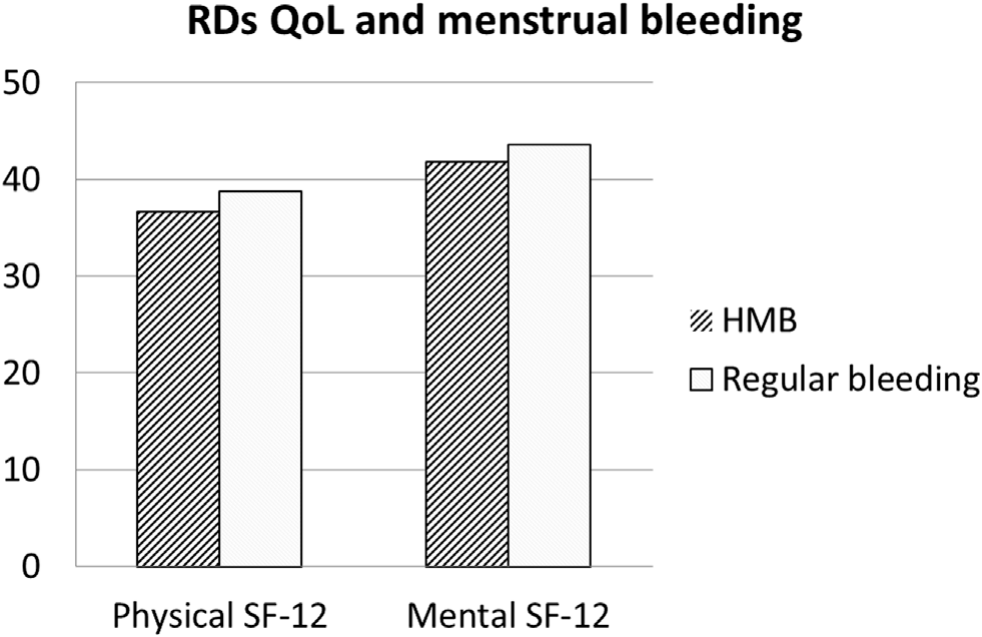
Quality of Life in RDs patients: Comparison between patients with regular bleeding and HMB. Legend: RDs: Rheumatic diseses.

**FIGURE 2:**
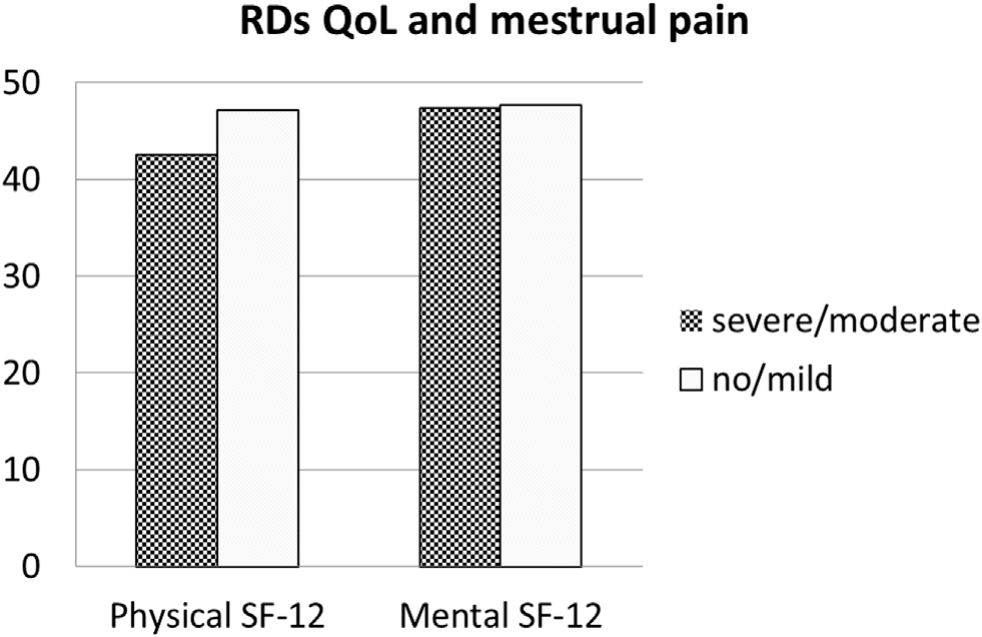
Quality of Life in RDs patients: Comparison between patients with dysmenorrhea and with normal or mild pain during menstruation. Legend: RDs: Rheumatic diseses.

The Qol evaluated with SF-12 among patients with RDs was lower both in mental domains (a physical component score (PCS-12) of 41.57 ± 12.47 and a mental component score (MCS-12) of 44.76 ± 11.23). In patients with RDs, comparing women with HMB vs normal bleeding, SF-physical and SF-mental were not statistically different, although a reduction in both QoL measures (SF-physical: 36.7 ± 16.2 and 38.8 ± 16.9, respectively; *p* = 0.470; SF-mental: 41.8 ± 14.5 and 43.6 ± 14.5) was respectively found (*p* = 0.468) (Figure 1). Similarly, SF-physical and SF-mental among women with RDs and with clinically significant dysmenorrhea was lower but not statistically significant with respect to women without dysmenorrhea (absent + mild) (SF-physical: 42.6 ± 22.6 and 47.2 ± 23.4, respectively; *p* = 0.1214; SF-mental: 47.4 ± 22.6 and 47.7 ± 23.3, respectively; *p* = 0.9113) (Figure 2).

### CTDs Group Versus Arthritis Group

On the basis of clinical and pathogenetic similarities, RDs were divided into a CTDs group and an arthritis group, but data were similar and no statistical differences were found in terms of menstrual pattern, gynecology, and fertility issues. Vasculitis was excluded because of the small sample size. Note that the family history of the HMB group (*p* = 0.506), miscarriages (*p* = 0.0286), asthma (*p* = 0.0193), and autoimmune thyroiditis (*p* = 0.008) were significantly more frequent in the CTDs group than in the arthritis group (Table 2).

### RDs Versus Control Group

Women with RDs reported more frequent HMB during adolescence (51.7 and 25.4%, respectively; *p* = .0001) and adult life (37.7 and 25.9%, respectively; *p* = .0065). Dysmenorrhea in adolescence was also more common (55.6 and 45.4%, respectively; *p* = .0338).

In all women with RDs, gynecological pain (dysmenorrhea, non-menstrual pelvic pain, dyspareunia, dysuria, and dyschezia) was significantly more frequent (*p* = .0001, .0001, .0001, .0001, and .0002, respectively) (Table 3). In particular, considering only moderate and severe symptoms, dysmenorrhea and dyspareunia were significantly higher in women with RDs with respect to controls (44.2 versus 21.3%, *p* = .0001 and 17.9 versus 8.1%, *p* = .0022; respectively) (Table 3).

**TABLE 3:** Gynecological data of rheumatic diseases vs controls: first 1,000 days of life, gynecological history, and menstruation-related disorders.

Gynecological data	RDs (n = 200)	Controls (n = 305)	p value
Age (years)	39.1 ± 8.7	35.8 ± 6.5	0.3764
BMI	23.3 ± 6.5	22.9 ± 5.5	0.4578
First 1,000 days of life			
Preterm birth	15/186 (8.0%)	16/301 (5.3%)	0.2612
Cesarean section	17/189 (8.9%)	37/302 (12.2%)	0.3756
Birth weight (gr)	3,269 ± 0.614	3,229 ± 514	0.4293
Formula feeding	70/189 (37%)	127/290 (43.7%)	0.1547
Gynecological history in adolescence			
Menarche	12.3 ± 2.0	12.4 ± 1.6	0.2146
HMB	101/195 (51.7%)	77/303 (25.4%)	0.0001*
Dysmenorrhea	108/194 (55.6%)	136/299 (45.4%)	0.0338*
Current characteristics of menstrual cycle			
HMB	71/188 (37.7%)	79/305 (25.9%)	0.0065*
Gynecological pain (mild + moderate + severe)			
Dysmenorrhea	130/183 (71%)	65/305 (21.3%)	0.0001*
Non-menstrual pelvic pain	49/187 (26.2%)	18/305 (5.9%)	0.0001*
Dyspareunia	72/184 (39.13%)	25/305 (8.1%)	0.0001*
Dysuria	35/188 (18.6%)	17/305 (5.2%)	0.0001*
Dyschezia	35/188 (18.6%)	23/305 (7.5%)	0.0002*
Gynecological pain (moderate + severe)			
Dysmenorrhea	81/183 (44.2%)	65/305 (21.3%)	0.0001*
Non-menstrual pelvic pain	6/187 (3.2%)	18/305 (5.9%)	0.2022
Dyspareunia	33/184 (17.9%)	25/305 (8.1%)	0.0022*
Dysuria	12/188 (6.3%)	17/305 (5.2%)	0.6987
Dyschezia	13/188 (6.9%)	23/305 (7.5%)	0.8600
Gynecological diseases			
Endometriosis	20/200 (10%)	24/305 (7.8%)	0.4231
PCOS	46/182 (25.27%)	79/305 (25.9%)	0.9148
Uterine fibroids	46/193 (23.83%)	61/305 (20.2%)	0.3157
Quality of life			
SF-physical	41.5 ± 12.4	51.2 ± 6.0	0.0001*
SF-mental	44.7 ± 11.2	47.6 ± 9.0	0.0014*

Legend: RDs, rheumatic diseases; HMB, heavy menstrual bleeding; PCOS, polycystic ovary syndrome; *p < 0.05.

No statistical significance was found comparing the gynecological comorbidities (PCOS, endometriosis, and uterine fibroids) between patients with RDs and the control group (Table 3).

**FIGURE 3:**
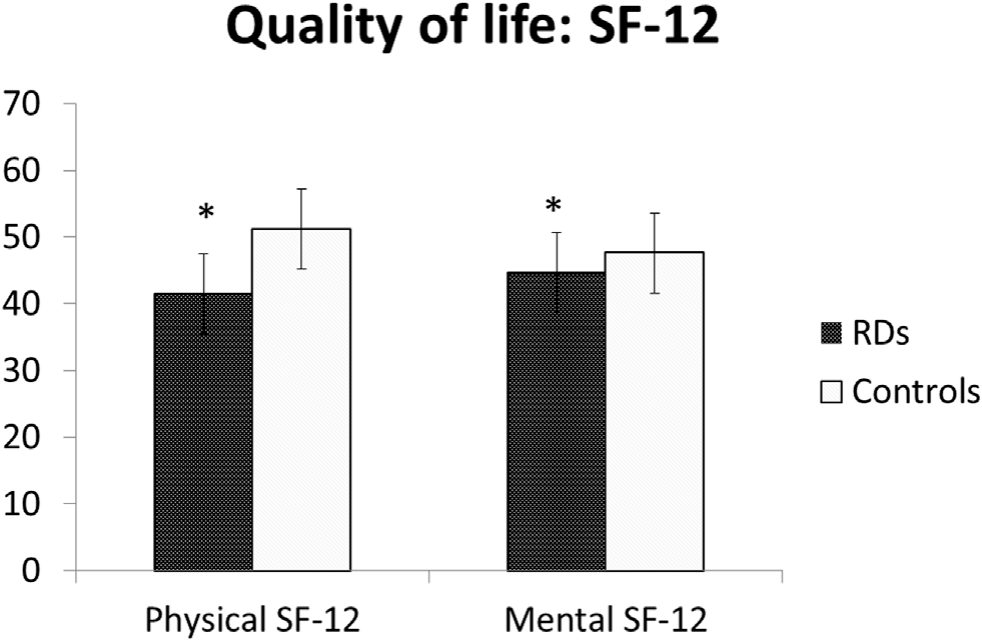
Quality of Life in RDs patients versus control group. Legend: RDs: Rheumatic diseases.

The Qol scores were significantly reduced among women with RDs, either in physical (41.5 ± 12.4 vs 51.2 ± 6.0, *p* = .0001) and mental domains (44.7 ± 11.2 vs 47.6 ± 9.0, *p* = .0014) of short-form 12 (SF-12) (Table 3; Figure 3).

## Discussion

The present study investigated the gynecological history, menstrual cycle pattern, menstrual-related symptoms/disorders, and QoL of women with RDs. At the best of our knowledge, this is the first study that extensively analyzed these aspects in fertile age women with RDs. So far, most pieces of evidence are available on fertility issues and pregnancy management of women with RDs (Østensen et al., 2015; Østensen, 2017), whereas menstruation-related disorders are often neglected. Indeed, menstruation-related disorders deeply affect QoL and women’s health, and for this reason, they should be considered in the daily clinical practice and care of women with RDs, who represent the majority of patients with RDs.

Menstruation is characterized by two physiological phenomena: pain (related to the uterine contractility) and bleeding (shedding of endometrial cells). According to their intensity and volume, they may become relevant issues for women’s health and QoL, other than early signs of uterine disorders.

Our data show that a significant number of women with RDs report menstrual disorders. In fact, dysmenorrhea, defined as pain during menstrual period, was found in more than half of the population since adolescence. Similarly, during fertile age, menstrual pain was frequently reported, and in 72% of cases, the intensity resulted moderate/severe. HMB, defined as abnormally heavy or prolonged menstrual bleeding, was found in 38% of our patients during adulthood and in 52% of patients during adolescence. In fertile age women, the prevalence of dysmenorrhea and HMB is thought to be high, but figures are not consistent as those symptoms are often considered “normal” by women, even though they may affect social life and work performances decreasing Qol. In our study, among patients with RDs, the Qol evaluated with SF-12 was lower both in physical and mental domains. Comparing women with HMB vs normal bleeding among patients with RD, SF-physical and SF-mental were reduced but did not reach the statistical significance. Similarly, SF-physical and SF-mental among women with RDs with significant dysmenorrhea were lower but not statistically significant than those of women without dysmenorrhea. Moreover, in some women, these symptoms may be associated with the development of a uterine disorder–like endometriosis or uterine fibroids. In the present study, uterine fibroma was reported by 24% of patients and endometriosis in 10%.

Gynecological pain is also another frequent symptom: 26% of patients referred non-menstrual pelvic pain, 19% urinary pain, and 18% dyschezia. Dyspareunia is a concurrent symptom in these patients, and its prevalence in patients with RDs is significant. Since these symptoms may represent early signs of gynecological diseases, such as endometriosis (Agarwal et al., 2019; Zondervan et al., 2020) and adenomyosis (Chapron et al., 2020), our data may be considered of outmost importance.

It is interesting to remark that menstrual pain and non-menstrual gynecological pain (dysmenorrhea, non-menstrual pelvic pain, dysuria, dyspareunia, and dyschezia) were significantly more frequent in women with RDs. Moreover, when only moderate and severe symptoms were considered (because reputed clinically more relevant), dyspareunia and dysmenorrhea remain significantly more frequent in women with RDs (Table 3).

Recently, some studies have shown a significant association between autoimmune diseases and endometriosis (Shigesi et al., 2019), which is a fertile age disease characterized by pain and infertility and caused by the ectopic localization of endometrial cells outside the uterine cavity, undergoing the same menstrual cyclic changes (Chapron et al., 2019). Frequently, the diagnosis is late, due to the heterogeneity of symptoms and the “normalization” of menstrual pain. Therefore, women affected by RDs should be considered a high-risk population for endometriosis, especially when a severe dysmenorrhea is reported.

In addition, women with RDs reported more frequent HMB during adolescence and adult life than controls. These findings also focus the attention on uterine fibroids and adenomyosis, uterine disorders characterized by HMB (Vannuccini et al., 2017), affecting both fertile age and perimenopausal women (Vannuccini and Petraglia, 2019). A previous study on women with primary Sjogren’s syndrome found a significantly higher prevalence of menorrhagia/metrorrhagia (Haga et al., 2005) (according to the old definition, it corresponds to HMB (Munro et al., 2018)) than controls. However, no prospective imaging studies have been performed on the incidence of adenomyosis or other uterine disorders, such as uterine fibroids, that may be associated with HMB. However, given the impact of HMB on QoL (Karlsson et al., 2014) and considering the consequences on women’s health, namely, anemia and fatigue (Percy et al., 2017), it seems important to investigate those symptoms in order to promptly refer the patient for further investigation in a gynecological setting.

The pathogenetic mechanism behind menstrual disorders among patients with RDs may be represented by the interrelationship between sex hormones and immunity (Zandman-Goddard et al., 2007). In fact, estrogens and progesterone are signaling modulators of the immune system, playing a role in lymphocyte maturation, activation, and synthesis of antibodies and cytokines (Ackerman, 2006). On the other hand, gynecological diseases such as endometriosis and adenomyosis have a strong immune background (Králíčková et al., 2018; Bourdon et al., 2021). Thus, immune dysregulation and inflammation may contribute to the development of menstruation-related disorders among women with RDs. Sex hormone expression is altered among patients with autoimmune disease, and this variation of expression contributes to immune dysregulation. Our results suggest that inflammation in RD could play a role in the pathogenesis of gynecological problems linked to menstruation (both dysmenorrhea and HMB). Therefore, when patients with RDs were stratified, no significant differences were found among two groups (CTDs and arthritis), suggesting that the origin of menstrual disorders may be due to both autoimmunity and inflammation. However, this could also mean that menstrual disorders are linked to gynecological alterations independent from the RD itself. Another hypothesis relies on the fact that these disorders may depend upon a hormonal imbalance determining an altered uterine contractility or to an endometrial issue with heavy bleeding (Critchley et al., 2020b).

In patients with RDs, gynecological sicca symptoms are commonly found, especially when Sjogren’s syndrome is present (MaddaliBongi et al., 2013; van Nimwegen et al., 2015). Our results confirm few data are present in the literature concerning vaginal symptoms: 36% of patients had vaginal dryness, 29% burning, 19% recurrent vaginal infections, and 39% dyspareunia (genital pain experienced before, during, or after sexual intercourse). The present study reported a higher prevalence of dyspareunia in RDs when than in controls, and the causality may be multifactorial: sicca syndrome, articular pain, muscular pain, psychological factors, and gynecological comorbidities (endometriosis, pelvic, and inflammatory disease). Dyspareunia has gone vastly underreported and untreated both in healthy subjects (Sobhgol and AlizadeliCharndabee, 2007) and RDs (MaddaliBongi et al., 2013). Therefore, since it may be a symptom of organic disease, it should be included in the clinical rheumatological evaluation to reach an early suspicion, thus referring the patient to a gynecologist for the diagnosis and a primary preventive care.

Confirming previous studies, Qol in patients with RDs was lower than that in control women, both in physical and mental domains of SF-12. However, it is well known that in patients with RDs, Qol is reduced due to various factors (drugs and comorbidities). In RDs referring to HMB, the subanalysis showed a lower Qol in women with HMB both in mental and physical domains. On the contrary, in the group with RDs, dysmenorrhea affected the SF-physical domain but not the SF-mental domain.

The weakness of our study may be found in the fact that patients affected by different RDs were enrolled and that the questionnaires were self-administered. However, the strength is also the innovative approach to gynecological problems in patients with RDs and also the high number of patients studied. Future studies should analyze the presence of polymenorrhagia and oligomenorrhea in patients with RDs and the possible role of RD therapy on menstrual disorders.

## Conclusion

Women with RDs show a high prevalence of various gynecological symptoms/disorders related to menstruation affecting their Qol. In particular, menstrual disorders are frequently detected in women with RDs, and this evidence should always prompt an investigation to exclude a concurrent gynecological disease. For this reason, the management of female patients with RDs is a challenge for clinicians and should include an accurate evaluation of gynecological aspects (menstruation, fertility, maternity, and sexuality) as well as manage an interdisciplinary teamwork approach (rheumatologist and gynecologists).

## Data Availability

The original contributions presented in the study are included in the article/Supplementary Material; further inquiries can be directed to the corresponding author.
