# Supplementary material for: Menstruation-Related Disorders—Dysmenorrhea and Heavy Bleeding—as Significant Epiphenomena in Women With Rheumatic Diseases
Source: Front Pharmacol. 2022 Feb 4;13:807880. doi: 10.3389/fphar.2022.807880 (PMC8854987; doi:10.3389/fphar.2022.807880)
Supplement: Supplementary file 1 [file DataSheet1.docx]

**SURVEY FOR PATIENTS WITH RHEUMATIC DIASEASE (RD)**

***Patient ID:***

**Age:** ________

**Weight (Kg):** ________

**Height (cm)**: ________

**Diagnosis of Rheumatic disease:**

□ Systemic lupus erythematosus

□ Rheumatoid arthritis

□ Systemic sclerosis

□ Sjögren’s Syndrome

□ Spondylitis Ankylosing

□ Psoriatic arthritis

□ Undifferentiated Connective tissue disease

□ Mixed connective disease

□ Overlap syndrome

□ Behcet

□ Seronegative arthritis

□ Churg Strauss syndrome

**Age at RD diagnosis:**

--------------

**RD duration:**

--------------

**Gynaecological family history:**

□ Heavy menstrual bleeding

□ Dysmenorrhea

□ Chronic pelvic pain

**First 1000 days:**

□ caesarean birth

□ pre-term birth

mean weight at birth (g): __________

□ breastfeeding

□ formula feeding

□ mixed feeding

**Gynaecological history in adolescence:**

Menarche in years: _________

□ Dysmenorrhea

□ Heavy menstrual bleeding

**Current characteristics of menstrual cycle:**

If the symptom is present, for each symptom, chose a number in the point scale from 0 (no pain) to 10 (maximum pain), based on to your proven experience.

The pain is considered as mild for 1-3 points, moderate for 4-7 points and severe for 8-10 severe pain.

□ Dysmenorrhea

If present:

0 5 10

______________________________________________

□ Heavy menstrual bleeding

If present:

0 5 10

______________________________________________

**Pain symptoms:**

 Non-menstrual pelvic pain

If present:

0 5 10

______________________________________________

 Dysuria

If present:

0 5 10

______________________________________________

 Dyschezia

If present:

0 5 10

______________________________________________

**Sicca symptoms and sexual dysfunction:**

 Vaginal dryness

If present:

0 5 10

______________________________________________

 Vaginal burning

If present:

0 5 10

______________________________________________

 Recurrent vaginal infections

 Dyspareunia

If present:

0 5 10

______________________________________________

If present:

 external vaginal pain

 deeper pain

 both

**Fertility and reproductive health:**

 time-to-pregnancy > 1 year

 Previous miscarriage/s

If yes, number:_________

 Previous pregnancy/s

If yes, number:_________

 Previous vaginal delivery

 Previous caesarean section

**Gynaecological comorbidities:**

 Uterine fibroids

 Endometriosis

 PCOS

**Comorbidities:**

 Other autoimmune disorders

 Autoimmune thyroiditis

 Endocrine/metabolic diseases

 Inflammatory bowel diseases

 Mental health disorders

 Depression

 Anxiety
